# Correlation between facial measurements and vertical dimension of occlusion among Tunisian populations: An anthropometric study

**DOI:** 10.34172/joddd.2022.014

**Published:** 2022-10-15

**Authors:** Imed Ouni, Rania Jebali, Sinda Amar, Lamia Mansour

**Affiliations:** ^1^Department of Prosthodontics, Dental Clinic of Monastir, University of Monastir, Tunisia; ^2^Private Dental Practitioner, Monastir, Tunisia

**Keywords:** Anthropometry, Craniometry, Ethnic groups, Occlusion vertical dimension

## Abstract

**Background.** Establishing an accurate occlusal vertical dimension (OVD) is a crucial clinical step during full-mouth rehabilitation. Various techniques have been suggested to evaluate OVD, but none of them is practically reliable, and each one has its shortcomings. The correlation between facial proportions and the lower third of the face is a reliable method but needs to be verified in many ethnic groups. Therefore, this study aimed to determine the correlation between OVD and various facial measurements in a Tunisian ethnic group.

**Methods.** A cross-sectional study was conducted between November 2020 to January 2021. The participants were randomly selected from dental students, dental surgeons, and the patients referring to the University Dental Clinic for dental treatments. Seven facial measurements were clinically recorded using a digital caliper. The correlation between OVD and facial measurements was analyzed using Spearman’s coefficient and linear regression analysis.

**Results.** A total of 201 dentate participants (134 females and 67 males) were included in the study. The mean OVD in male subjects was higher (67.60±4.49) compared to female subjects (60.72±3.84). The total facial height was positively correlated with OVD in both genders. OVD was statistically correlated with the height of the upper lip. This correlation was highly significant in males while it was weak in the female group.

**Conclusion.** Facial proportions and linear equations are non-invasive, simple, and reliable methods to predict OVD, especially in males.

## Introduction

 The Glossary of Prosthodontic Terms defines the occlusal vertical dimension (OVD) as the distance between two marked anatomic points (generally between the gnathion and the tip of the nose) in the maximal intercuspal position.^1^ Incorrect OVD adversely affects the occlusal stability, the masticatory muscles, aesthetics, and functional efficiency. Moreover, the inappropriate height of the lower third of the face leads to facial expression disabilities and problems in the rest position in geriatric patients.^2^

 Although many methods exist to determine OVD, none of them is practically reliable, and each one has its shortcomings.^3^ Various parameters have been suggested to be responsible for the ambiguities in the registration of OVD, including variability in pathologic and physiologic conditions of the edentulous patient.^4^ The inaccuracy of these techniques makes the registration of OVD subjective, sometimes requiring an ‘individual clinical judgment of the dentist.

 Some researchers recommend using anthropometric measurements to determine OVD: Ladda et al^5^ found a correlation between the length of fingers and original OVD. Similarly, another study found a significant association between OVD and interpupillary distance at a range of 2–4 mm in male patients.^3^ These anthropometric methods are non-invasive, simple, and cost-effective and could be used for everyday practice.

 The generalization of the correlation between OVD and facial measurements requires further studies, including differences in racial and ethnic groups and gender dimorphism.^6^ Anthropometric measurements in the local population will help practitioners during OVD rehabilitation. Investigations into the correlation between OVD and craniofacial distances in the Tunisian population are rare or even absent in the dental literature.

 Hence, this study evaluated the correlation between OVD and facial landmark measurements in a North African ethnic group.

## Methods

 This descriptive cross-sectional study was conducted from November 2020 to January 2021 in the Department of Prosthodontics, Faculty of Dentistry, Monastir, Tunisia. The study was approved by the institutional ethics committee. Participants were randomly selected from postgraduate and undergraduate dental students, dental surgeons, and the patients referring to the University Dental Clinic for dental treatment. The participants were informed about the objectives and methodology of the study, and written consent was obtained.

 All the participants were < 30 years of age with fully erupted permanent teeth (third molars were not taken into account) and a normognathic facial profile. Subjects with previous prosthodontic or orthodontic treatment, facial asymmetry, deformities and scars, temporomandibular joint disorders, and extensive stomatognathic surgery were excluded.

**Figure 1:**
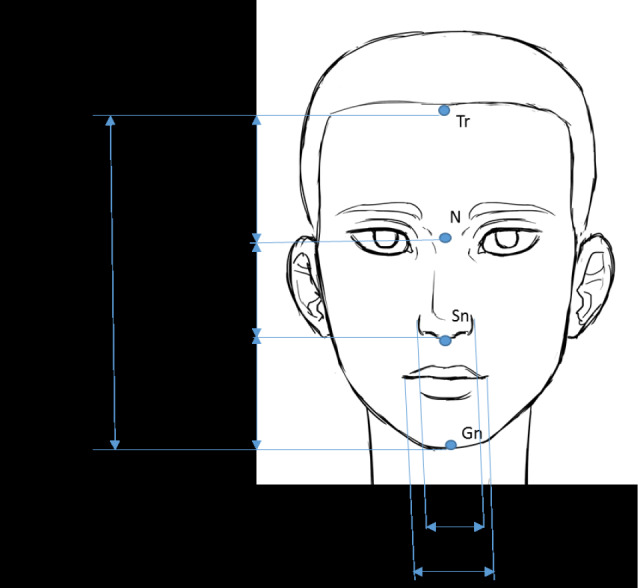
Various facial measurements: Tr,Trichion point; N, Nasion point; Sn, Subnasal point; Gn,Gnathion

 To have fixed landmarks, the participants were asked to sit upright with the head vertical and unsupported and gaze fixed on the horizon. Indeed, it is a reference position to have the balance between bone and muscular elements described in the classic diagram of Brodie and recommended during the determination of OVD in complete dentures.^7^ The facial measurements were recorded using a digital caliper and a graduated ruler. An indelible pencil marked the soft tissue points on the face. The following facial measurements were registered during the study^8,9^ (Figure 1):

- Total facial height (TFH) was defined as the distance between the trichion and the gnathion.
- The upper third of the face (UFH) was measured between the trichion and the nasion.
- The middle third of the face (MFH) was determined between the nasion and the subnasal point.
- The height of the lower third of the face in maximal intercuspal position or OVD was measured between the gnathion and the subnasal point.
- The height of the upper lip (ULH) was measured between the subnasal point and the stomion point.
- The inter-nostril distance (IND) represents the width of the base of the nose.
- The inter-commissural distance (ICD) was measured between the two corners of the mouth

Facial measurements were recorded in millimeters. The data analysis was performed using SPSS 21. The level of significance was determined at *P* < 0.05. The correlation between OVD and facial measurements was evaluated by Spearman’s correlation coefficient. A regression analysis was carried out to determine the linear equation to predict the OVD.

## Results

 A total of 201 participants, consisting of 134 females and 67 males, were included in the study. The mean value of OVD for males was 67.60 (4.49) mm, with 60.72 (3.84) mm in females. The Student’s *t* test was used to compare the mean OVD, which showed a significant difference between male and female participants (t = 11.292, *P* ≤ 0.001) (Table 1).

**Table 1:** Student’s *t* test to compare facial measurements and OVD in male and female subjects

	**Mean (SD)**		**T**	***P***
	**Female (n=134)**	**Male (n=67)**		
TFH	181.52 (10.30)	195.90 (11.22)	9.050	0.000
UFH	65.50 (50.43)	65.90 (6.14)	0.063	0.475
MFH	57.80 (4.43)	60.75 (5.46)	4.106	0.000
OVD	60.72(3.84)	67.60 (4.49)	11.292	0.000
ULH	22.59 (18.21)	21.12 (2.14)	-0.658	0.255
IND	32.37 (2.32)	34.61 (3.31)	5.579	0.000
ICD	47.25 (3.15)	48.18 (3.25)	1.957	0.26

T-test. *P *value < 0.05, significant correlation.

 Spearman’s correlation coefficient was used to determine the correlation between OVD and other facial measurements (Table 2). The results showed that OVD was positively correlated with some measurements studied in male and female groups.

**Table 2:** Correlation by Spearman’s test between OVD and other facial measurements in both gender

	**Male (n=67)**		**Female (n=134)**	
	***r***	***P***	***r***	***P***
TFH	0.669	0.000	0.572	0.000
UFH	0.468	0.000	0.147	0.087
MFH	0.038	0.758	0.119	0.167
ULH	0.587	0.000	0.184	0.032
IND	0.316	0.009	0.14	0.875
ICD	0.160	0.197	0.159	0.064

r: Spearman’s correlation coefficient; *P* value < 0.05, significant correlation; *P* < 0.001, highly significant correlation.

 A highly significant correlation was observed between OVD and TFH in both genders (r = 0.669 for males and r = 0.572 for females). OVD was significantly correlated to the height of the upper lip (ULH). This correlation was highly significant in males (*P* ≤ 0.001 and *r* = 0.587), while it was weak in the female group (*P* = 0.032 and *r* = 0.184).

 A moderate positive correlation was found between OVD and UFH (*P* ≤ 0.001 and *r* = 0.468), OVD and IND (*P* = 0.009 and *r* = 0.316), and OVD and IPD (*P* = 0.036 and *r* = 0.257) only in male subjects.

 After finding the correlation between OVD and some facial indexes, a regression analysis was carried out to predict OVD using facial measurements and obtain the linear equations of regression (Table 3). For male subjects, the following linear equations were reliable to determine OVD:

 - OVD = 15.14 + 0.268*TFH

 - OVD = 45.02 + 0.343*UFH

 - OVD = 41.59 + 1.232*ULH

 - OVD = 52.75 + 0.429*IND

 For females, only the following linear equation was reliable to determine OVD:

 - OVD = 22.03 + 0.213*TFH

**Table 3:** Regression analysis for variables predicting OVD

	**Male (n=67)**				**Female (n=134)**			
	**r** ^2^	**B**	**SEB**	***β***	**r** ^2^	**B**	**SEB**	***β***
TFH	0.447	0.268	0.037	0.669*	0.327	0.213	0.027	0.572*
UFH	0.219	0.343	0.080	0.468*	0.023	0.012	0.007	0.153
MFH	0.001	0.032	0.102	0.038	0.013	0.097	0.075	0.112
ULH	0.345	1.232	0.210	0.587*	0.035	0.040	0.018	0.188
IND	0.100	0.429	0.160	0.316*	0.000	-0.013	0.144	-0.008
ICD	0.026	0.221	0.169	0.16	0.025	0.191	0.105	0.157

r^2^: coefficient of determination; B: standardized beta; SEB: standard error for B; β: standardized beta (*P < 0.05).

## Discussion

 Leonardo Da Vinci was the first to correlate OVD with various anthropometric measurements to make accurate drawings. Later explorations by researchers were followed to find the gold proportion between OVD and other facial measurements in many ethnic groups.^4,10^ Since there are notable phenotypic and genotypic variations between different ethnic groups,^11^ it is important to investigate the hypothesis in various populations. Therefore, this study was conducted on a Tunisian population to evaluate the correlation between OVD and facial measurements.

 The mean OVD registered in male subjects of this study was similar to the results recorded in studies on a Saudi Arabian population (69.25 ± 5.54),^10^ on Iraqi males (68.25 ± 6.13)^12^ and on Angolan (67.3 ± 4.8 mm) and Germans men (67.9 ± 7.3 mm) described in an international anthropometric study.^11^ However, the mean value of OVD in an Indian male population was lower (61.4 ± 4.2).^3,5^ The current study also showed a similar mean OVD in the female group (60.72 ± 3.84) compared to Turkish (59.1 ± 3.8) and Polish females (60.5 ± 4.4) and slightly lower than the value recorded in Angolan women (63.2 ± 6.6).

 The similarity of the results with different ethnic groups could be explained by the strategic geographic position of Tunisia, which is located at the crossroads of Europe, the Middle East, and sub-Saharan Africa^13^ as well as a complex demographic history of migrations.^14,15^

 The majority of facial measurements were significantly higher in males than in female subjects; the difference was attributed to sex-related characteristics of maxillofacial growth.^16^

 A strong positive correlation was observed between OVD and TFH in both genders. Ricketts^17^ reported the OVD value as a function of the total facial height; the slope of the regression line was 0.25, which is very similar to our study (the slopes of the regression lines were 0.268 and 0.213, respectively for males and females).

 The regression analysis of OVD based on the height of the upper third of the face (UFH) showed equality between the two upper and lower levels of the face in male subjects, a conclusion already drawn by Ricketts,^17^ who considered the middle third of the face as a zone of balance or congruence guaranteeing the maintenance of facial proportionality. Similar results were found in a Saudi Arabian population.^10^ Thus, the UFH can be an interesting reference for determining accurate OVD in male patients.

 The current study showed a significant correlation between OVD and the height of the upper lip (ULH) in males. The slope of the regression line was 1.232. This ratio was previously mentioned by Farkas et al.^18^ Similar findings were reported by Mbodj et al^19^ in a study on young subjects with young melanoderm subjects and by Kassab et al.^20^ in a study on young Caucasian subjects.

 However, this correlation was weak in females (r = 0.184), which might be explained by the rapid and early onset of the aging process in women, leading to significant variability in the values of ULH recorded in the female population. Hence, it is not recommended to predict OVD due to the important inter-sex variability.

 A positive correlation was found between OVD and IND only in males (*P* = 0.009 and *r* = 0.316). The slope of the regression line was 0.429. It is, therefore, possible to predict the value of OVD from the nasal width in male patients.

 The limitation of the study was that only subjects with class I malocclusion were included; therefore, the correlation in other dental and skeletal malocclusions requires further research. The participants were from the native population; hence, the regression formula and results are not suitable for other ethnic groups.

## Conclusion

 The total facial height is a reliable measurement to predict OVD for both genders in the Tunisian population. Other facial measurements like the height of the upper face, the height of the upper lip, and the width of the base of the nose can be used in males as a measurement for OVD approximation. Further investigations are required to confirm the correlation of OVD with facial measurements in various ethnic groups.

## Acknowledgments

 None.

## Authors’ Contributions

 IO and RJ: conception and design. SA and LM: acquisition, analysis, and interpretation of data. All authors have read and approved the final manuscript.

## Funding

 No funding sources.

## Ethics Approval

 The study was approved by the institutional ethics committee in the Faculty of Dentistry, Monastir, Tunisia.

## Competing Interests

 No conflicts of interest.
